# Using Implementation Mapping to develop and test an implementation strategy for active learning to promote physical activity in children: a feasibility study using a hybrid type 2 design

**DOI:** 10.1186/s43058-022-00271-9

**Published:** 2022-03-07

**Authors:** Timothy J. Walker, Harold W. Kohl, John B. Bartholomew, Charles Green, Maria E. Fernández

**Affiliations:** 1 Department of Health Promotion & Behavioral Sciences, Center for Health Promotion and Prevention Research, The University of Texas Health Science Center at Houston School of Public Health, 7000 Fannin St., TX 77030 Houston, USA; 2 Department of Epidemiology, Human Genetics and Environmental Sciences, The University of Texas Health Science Center at Houston School of Public Health, Austin Regional Campus, 1616 Guadalupe, Austin, TX 78701 USA; 3 Department of Kinesiology and Health Education, The University of Texas at Austin, 2109 San Jacinto Blvd, Austin, TX 78712 USA; 4 Department of Kinesiology and Health Education, The University of Texas at Austin, 2109 San Jacinto Blvd, Austin, TX 78712 USA; 5 Department of Pediatrics, Center for Clinical Research and Evidence-Based Medicine, The University of Texas Health Science Center at Houston McGovern Medical School, 6431 Fannin Street, Houston, TX 77030 USA

**Keywords:** Implementation strategies, Active learning, Physical activity, Implementation science, Implementation Mapping

## Abstract

**Background:**

Incorporating physical movement in the teaching of academic content (active learning) is a promising approach to improve children’s health and academic performance. Despite documented benefits, implementation of active learning remains challenging for schools. The aims of this study are to develop an implementation strategy to support the delivery of active learning in elementary schools and examine the impact of the developed implementation strategy on the implementation and effectiveness of active learning.

**Methods:**

Aim 1 will use Implementation Mapping, which is a multi-step approach that guides the use of theory, stakeholder input, and existing literature to develop a scientifically based implementation strategy for active learning in elementary schools. Aim 2 will feature a feasibility study to examine the impact of the implementation strategy on both implementation and effectiveness outcomes, consistent with a Hybrid Type 2 design. Acceptability and implementation fidelity will be the primary implementation outcomes, and student physical activity levels will be the primary effectiveness outcome. We will recruit two elementary schools within our partner district, and one will be randomly assigned to receive usual support while the other will receive the newly developed implementation strategy. Participants from each school will complete baseline, 6-, and 12-month assessments. Bayesian statistical approaches will quantitatively examine preliminary effectiveness outcomes. We will also use an embedded mixed methods approach to triangulate findings.

**Discussion:**

This study’s innovative overarching conceptual framework (centered on Implementation Mapping) will inform the development and testing of the implementation strategy. This study also uses methodological approaches optimal for feasibility studies, including mixed methods and Bayesian statistics. As a result, we will be able to gain a thorough understanding about the feasibility and preliminary effectiveness of the implementation strategy, which will inform subsequent research and practice for implementing active learning in schools.

**Trial registration:**

ClinicalTrials.gov, NCT05048433, registered on September 8, 2021.

Contributions to the literature

- This study uses a systematic approach, Implementation Mapping, to develop an implementation strategy for active learning in elementary schools.
- This study describes a comprehensive evaluation plan for a feasibility study that includes the use of Bayesian statistics and mixed methods.
- The implementation strategy development and evaluation approach can serve as an example approach to better understanding preliminary effectiveness and potential mechanisms through which implementation strategies operate

## Background

About 49% of boys and 36% of girls (aged 6–11 years) are meeting physical activity guideline recommendations [1]. As low levels of physical activity in childhood track into adulthood, this puts a generation of children at an increased risk for cardiovascular disease and other chronic conditions. The American Heart Association recommends population-based physical activity approaches that start in early childhood for cardiovascular disease prevention [2]. Despite existing evidence-based approaches that increase children’s physical activity, implementation remains a challenge, especially in settings such as schools.

With the increased emphasis on standardized test scores, school administrators often devote more time throughout the school day to reading and math and less time to physical education and recess [3]. One way in which educators are adding physical activity opportunities back into the school day is through active learning approaches, which incorporate physical movement into academic lessons. Research indicates that active learning increases physical activity levels of students [4, 5] and can add about 19 min of moderate-to-vigorous intensity activity to the school day [6]. There is also evidence active learning decreases sedentary time [7], improves health fitness outcomes [8, 9], and improves on-task behavior [10–12].

Despite the promise of active-learning, these approaches are inadequately implemented to maximize the public health benefits [13, 14]. In a nationally representative sample of 640 elementary schools, 75% of schools reported using classroom-based physical activity approaches [14]. However, schools serving predominantly economically disadvantaged or minority students were 43% and 52% less likely to use these approaches (respectively). Among schools using classroom-based physical activity approaches, only 45.6% of teachers used them regularly [14]. Studies have indicated multilevel barriers to implementation, including lack of time, low levels of knowledge about classroom-based approaches, and low perceived benefits at the individual level, and, a poor implementation climate, competing priorities, lack of space, and lack of administrative support, at the school level [15–18]. There is a pressing need to develop effective implementation strategies to address barriers and improve implementation of active learning.

Implementation strategies are methods or techniques that enhance the adoption, implementation, and sustainment of a program or practice [19]. Implementation strategies can be single (discrete) approaches, multifaceted strategies, or blended [20]. Discrete involve one action (e.g., a staff-wide training), multifaceted combine two or more discrete strategies, and blended are comprised of multiple discrete strategies that have been protocolized [20]. Recent studies suggest staff training is a common (yet often insufficient) discrete strategy used by schools to support classroom-based physical activity approaches [18]. Other promising discrete strategies include the use of program champions, professional learning communities, positive reinforcements, and strategic planning [18, 21, 22]. Despite the many discrete strategies, there has been little effort to systematically study multifaceted implementation strategies to understand how they can support the implementation and sustainment of classroom-based physical activity approaches.

This study focuses on improving the implementation and scale-up of active learning in schools to support student’s health and well-being. Study aims include the following: (1) develop an implementation strategy to improve the use and sustainment of active learning in elementary schools and (2) conduct a feasibility study to evaluate the impact of the developed implementation strategy on the implementation and effectiveness of active learning.

## Methods

### Theoretical framework

**Fig. 1:**
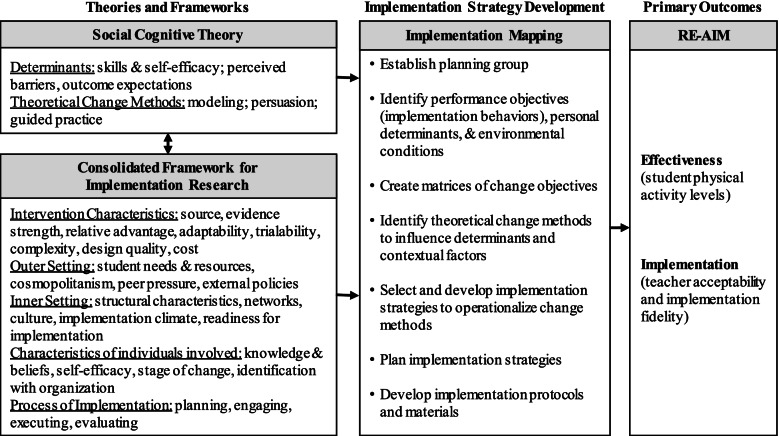
Conceptual framework

Due to the complex nature of implementation strategy development and evaluation, our work is guided by a comprehensive conceptual framework centered on Implementation Mapping (Fig. 1). Implementation Mapping [23] is a step-by-step process that guides the use of theory, empirical evidence, and stakeholder input for implementation strategy development. We also draw from Social Cognitive Theory (SCT) [24] and the Consolidated Framework for Implementation Research (CFIR) [25]. SCT permits specification of personal determinants and predictive relations driving implementation behaviors, while CFIR guides what behaviors and contextual factors are important to consider and target as part of the implementation strategy. Last, RE-AIM will guide evaluation by defining implementation and effectiveness outcomes [26]. This framework allows us to target a highly relevant public health issue through a theoretically informed, evidence-based process that prioritizes the important contextual factors of implementation in the school setting.

### Aim 1: Develop an implementation strategy for active learning

We will use Implementation Mapping to develop a multifaceted implementation strategy for active learning in elementary schools. As an initial step, we will establish a planning group that consists of teachers, school staff, school wellness professionals, and parents. This planning group will work with the research team throughout the project to support the development and evaluation of the implementation strategy.

**Fig. 2:**
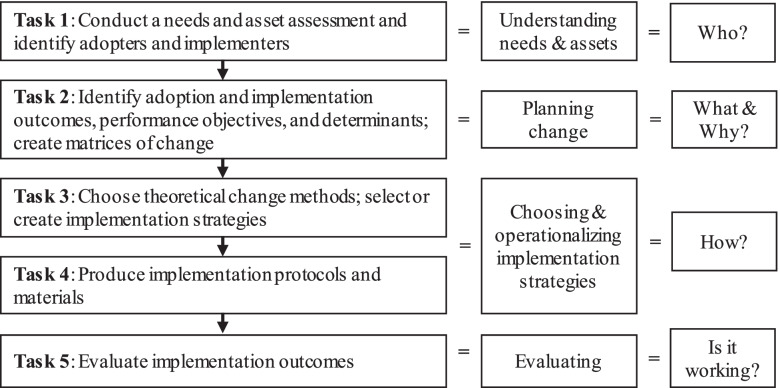
Implementation Mapping Tasks

Implementation Mapping consists of five tasks (Fig. 2). The first task is to conduct a needs and assets assessment to identify program adopters and implementers, and, potential barriers and facilitators to implementation. As part of our formative work, we conducted qualitative interviews and surveys with school staff to understand how physical activity programs are adopted [27], current implementation strategies used by schools [22, 28], and teacher- and school-level factors associated with implementation of classroom-based physical activity approaches [29–31]. We will expand on our formative work by collecting additional information about the specific needs and assets of schools within our partner district. The existing formative work, newly collected information, and input from our planning group will help specify those involved with implementation and the barriers and facilitators to implementation.

For Task 2, we will first identify implementation outcomes and performance objectives, which are the implementation behaviors required to achieve an implementation outcome. Performance objectives help specify “who has to do what?” to implement active learning approaches. Subsequently, we will identify relevant personal determinants and other contextual factors influencing implementation behavior. Determinants and contextual factors will help identify *why* a teacher would implement active learning approaches as planned. We will use the performance objectives, determinants, and contextual factors to create matrices of change objectives, which will specify the changes required in each determinant to achieve the corresponding implementation behavior.

We will use the matrices of change objectives to inform Task 3, which consists of selecting theoretical change methods and designing implementation strategies that operationalize those methods. Theoretical change methods are techniques to influence determinants of implementation (e.g., using guided practice to influence self-efficacy for implementing active learning approaches). Theoretical methods will focus on both personal determinants (e.g., self-efficacy) and organization-level contextual factors (e.g., implementation climate). As part of Task 3, we will design and develop a multifaceted implementation strategy (consisting of multiple discrete strategies) that will operationalize the identified theoretical methods. Task 4 involves developing implementation protocols and materials by creating design documents, drafting content, pretesting/refining content, and finalizing materials. The final Task of Implementation Mapping (Task 5) includes evaluating implementation outcomes. This is the focus of Aim 2 for this study.

### Aim 2: Conduct a feasibility study to evaluate the implementation strategy

#### Study design

The goal of the feasibility study is to determine whether the implementation strategy is appropriate for further testing [32]. The study is designed to determine (1) whether using an implementation strategy *can* improve delivery of active learning in schools and (2) whether there is evidence that the specific active learning approaches *will* increase student’s physical activity levels. By concurrently testing implementation and effectiveness outcomes, we are using an approach consistent with a Hybrid Type 2 design [33].

**Fig. 3:**
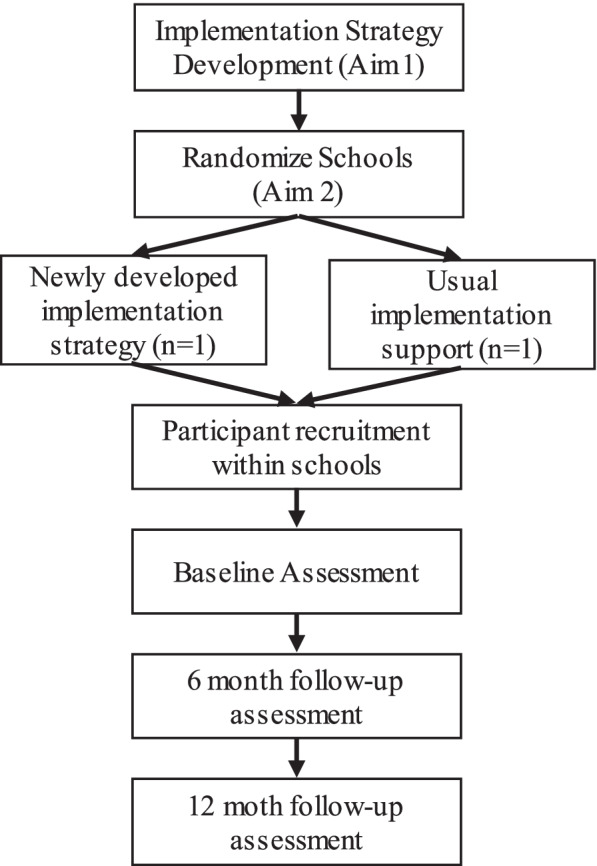
Flow diagram of study

We will recruit two elementary schools serving similar student populations and not formally implementing active learning approaches. We will randomly assign one school to receive the developed implementation strategy, and the other to receive usual implementation support (Fig. 3). Drawn from the same district, both schools will operate under similar broad policy and administrative support for active learning. Each school will complete baseline, 6-, and 12-month assessments. We will use an embedded mixed-methods approach by collecting quantitative and qualitative data to triangulate findings across implementation outcomes.

#### Setting and participants

Each school will receive $1000 for their participation in the study. Our partnering district serves an economically and culturally diverse community (>50% of students who are minority and >50% of students who are economically disadvantaged). We will recruit kindergarten-5th grade teachers from each school to participate in the study. Each teacher will be asked to complete assessments at each timepoint (baseline, 6, and 12 months) for which they will receive a $40 gift card. We will recruit a subsample of teachers (*n*≈12) to participate in semi-structured interviews for which they will receive a $25 gift card.

We will recruit students from each school to examine student-level outcomes. All students will be able to participate in active learning lessons, although for study participation, students must be able to speak English or Spanish and have no physical limitations. We will ask participating students to complete study questionnaires and to allow the school to share deidentified academic and health-related fitness data. We will randomly select a subsample of 100 students at each school to collect device-based physical activity data. We will use a stratified sampling method based on grade and classroom to ensure there is an equal proportion of students across grades and classrooms. Additionally, we will recruit a subsample of about 12 students to participate in semi-structured individual interviews. We will use a purposeful sampling method to recruit 6 boys and 6 girls, with at least one boy and girl from each grade (K-5). Students who participate in the interview will receive a $25 gift card.

#### Recruitment

We will work with our stakeholder planning group to inform study recruitment. We plan to use a mix of passive and proactive recruitment approaches to enroll study participants. Specifically, we will conduct in-person presentations at school staff meetings to explain procedures and enroll teachers in the study. We will also distribute study information packets to each student consisting of a general study flyer, a plain language consent form, and a child assent form. The packets will be available in English and Spanish. We will also present the project to students and families at back-to-school nights and existing school-sponsored events designed to engage hard-to-reach parents (e.g., parent education events). We will use recruitment incentives for students (e.g., pencils and erasers) and teachers (e.g., project t-shirts for teachers if 80% of students return consent forms from a class).

#### Data collection

There will be multiple forms of data collection including paper and pencil surveys for students, electronic surveys for teachers, implementation logs, direct observation, device-based physical activity assessment, and the use of existing data. Table 1 provides and overview of study variables, the method of assessment, and the collection time point. We will use Qualtrics to distribute the electronic survey link to teachers.

**Table 1:** Data collection

Variables	Assessment method	Time
**Primary outcomes**		
Acceptability^*I*^	Teacher and Student Surveys and Interviews	B and F/U
Implementation Fidelity^*I*^	Teacher Survey, Implementation log, Observation	F/U
Student Physical Activity^*E*^	Accelerometers and Youth Activity Profile	B and F/U
**Secondary outcomes**		
Health-Related Fitness	FitnessGram® (collected by schools)	F/U
Academic Performance	Test Scores (collected by schools)	B and F/U
Student Behavior	Office referrals (collected by schools)	B and F/U
**Additional variables**		
Demographics	Teacher Survey, Student data from district	B
CFIR and SCT constructs	Teacher Survey	B and F/U

^*I*^Implementation outcome; ^*E*^Effectiveness outcome; *B* baseline, *F/U* 6- & 12-month follow-up, *CFIR* Consolidated Framework for Implementation Research, *SCT* Social Cognitive Theory

Qualitative data collection will consist of semi-structured individual interviews with about 12 teachers and 12 students beginning after the 6-month follow-up assessment. The teacher interviews will include questions about the acceptability, fidelity, and sustainment of active learning. Student interviews will include questions about acceptability of active learning and physical activity in school. Teacher interviews will last 45–60 min, and student interviews will last about 30 min. All interviews will be recorded and transcribed for analysis.

#### Study outcomes

The RE-AIM framework will guide assessment of implementation and effectiveness outcomes [26]. Primary study outcomes will include *acceptability*, *fidelity*, and *student physical activity levels*. *Acceptability* is the perception that an intervention is agreeable, feasible, or satisfactory to recipients and implementers [34]. We will use the Acceptability of Implementation Measure (AIM) [35] to examine acceptability of the implementation strategy and active learning among teachers. We will adapt the AIM to examine acceptability of active learning among students [36]. We will also conduct teacher and student interviews to examine acceptability as previously described.

*Fidelity* is the degree to which an approach was implemented as prescribed and is typically measured in terms of adherence, dose of delivery, and quality of delivery [34]. As part of the implementation strategy development process, we will work with the planning group to articulate a definition of implementation fidelity to inform measurement. We will use self-reported implementation logs to assess dose, and direct observation and a self-reported questionnaire to assess quality and adherence [37]. We will also examine fidelity as part of the qualitative interviews. Implementation logs will be completed weekly for a 1-month span during the follow-up assessments. Direct observation will be conducted by trained staff using procedures consistent with previous research [38]. Staff will randomly select classrooms at different times of day during a 1-week span to track implementation quality and adherence. Ratings will be based on (1) how many children were active, (2) how often children were active, (3) the intensity of movement, and (4) adherence to core elements.

We will assess *student physical activity levels* using Actigraph GT3X+ accelerometers. We will examine minutes spent in moderate and vigorous physical activity in addition to other physical activity variables (e.g., sedentary time, total counts). Accelerometers will be worn on an elastic belt placed above the iliac crest of the hip of each participant [39, 40]. Each belt will be attached to students within 30 min of the start of school and removed within 30 min of dismissal. Given the variation in daily physical activity levels for children, we will use sampling epochs set at 5 s intervals to best capture this variability [41]. We will use cut points appropriate for children to classify time spent in respective activity intensities [42]. Children will wear accelerometers for 5 consecutive school days [43, 44].

Secondary outcomes include health-related fitness, academic performance, and student behavior. Health-related fitness variables include data from FitnessGram® assessments, which are completed annually for 3rd–5th grade students. We will use data collected from the previous year to serve as a baseline measure. We will examine body mass index (from height and weight measures) and aerobic capacity (from the 20-m PACER test) [45]. We will examine academic performance as an additional secondary outcome using growth tests aligned with the Texas Essential Knowledge and Skills standards. We will examine student behavior using office referral data collected by schools.

In addition to primary and secondary outcomes, we will also collect student demographic information (e.g., sex, age, race, qualifying for free/reduced cost lunch) from school records and student’s self-reported physical activity data using the Youth Activity Profile [46, 47] to assess in and out of school activity. We will also collect teacher demographic data (e.g., sex, age, race/ethnicity, number of years as a teacher, type of teacher, and subject) and assess variables from CFIR and SCT as part of the teacher survey. CFIR variables will include inner setting domain constructs: culture, implementation climate, learning climate, leadership engagement, and available resources [48, 49]. SCT variables will include skills, self-efficacy, attitudes, barriers, and outcome expectations [50]. Demographic variables will serve as potential confounders, and CFIR and SCT variables will be considered as mediators and moderators.

#### Data analysis and power

We will use generalized linear models to compare implementation fidelity and acceptability between teachers who received the strategy versus teachers who did not. Longitudinal/ repeated measures analyses will be conducted for student level variables using generalized linear multilevel modeling. Multilevel effects will address classroom clustering. We will use Bayesian approaches to implement joint modeling of observed outcomes and missing data, which is robust to ignorable missingness [51]. Convergence of Bayesian analyses on the posterior distributions via Monte-Carlo Markov chain will be assessed via graphical (Trace Plot, Autocorrelation Plot) and quantitative evidence (Gelman-Rubin Diagnostics and Effective Sample Size Diagnostics). Evaluation of posterior distributions will permit statements regarding the probability that effects of varying magnitudes exist, given the data. Specification of weak, neutral priors, and the prior distribution for level two variances will follow recommendations by Gelman [52, 53]. The impact of the implementation strategy on implementation and student outcomes will be based on the posterior distribution of effect sizes.

Given this is a feasibility study, the study purpose is to examine acceptability and effectiveness trends. Thus, the study is not designed to have the statistical power to examine school level differences in implementation outcomes. The study will provide important descriptive data and inform future work. Our analytic approach, applying Bayesian methods, will result in a posterior distribution for the credible effect size estimates, importantly providing both an index of the effect size and its associated uncertainty. This posterior distribution will permit probabilistic estimates of the alternative hypothesis. Moreover, subsequent trial planning will incorporate the entire posterior, both its estimate and associated uncertainty in developing a robust plan for a subsequent R01 study [54, 55].

## Discussion

This study contributes to the fields of implementation science and physical activity promotion in multiple ways. First, it uses an innovative overarching conceptual framework to inform the development of an implementation strategy to help schools implement active learning. Implementation Mapping (which is at the center) facilitates the link between health behavior theories and implementation frameworks, stakeholder input, and empirical evidence to ensure the developed implementation strategy is evidence-informed and appropriate for schools, staff, and students [23]. Further, the Implementation Mapping protocol links implementation behaviors and their determinants to theoretical change methods, in a manner that can help specify a complete (and testable) mechanism of action. Understanding the mechanisms for how implementation strategies impact outcomes is priority for the field and thus, this study can serve as an example for other studies developing and testing implementation strategies [56].

Another contribution of this study is the use of an evaluation plan and analytic approach that are optimal for feasibility studies. Feasibility studies play in an important role in helping determine whether an intervention (and in this case implementation strategy) should undergo further testing [32]. This feasibility study includes a mixed methods evaluation to gain a thorough understanding of the implementation strategy’s feasibility and preliminary effectiveness. The study also uses Bayesian statistical methods, which offer many advantages when working with small sample sizes and clustered data, which are common challenges for implementation studies. Results from this study will provide information about whether (and how) the developed implementation strategy influenced teacher’s implementation behaviors for active learning, as well as inform future planning efforts for larger scale research in this area.

There are several practical challenges for this work. Schools have been forced to adapt their approaches to maintain student health and safety during the COVID-19 pandemic [57]. Notably, schools have had to make abrupt systematic changes to maintain student learning, meet student needs, and respond to the concerns of their communities. Schools have shifted approaches for teaching, providing food services, and providing mental health support to students. Given the many changes due to the pandemic, schools may be reluctant to participate in a research study about active learning and teachers may experience additional implementation barriers during this time. We have been working closely with our district partner to maintain open communication, to offer flexibility for research tasks (e.g., flexible start dates and data collection schedules), and to expand the implementation development process to account for additional challenges imposed by the pandemic.

## Summary

The link between health and academics is well-established [58], yet given the many challenges schools face, health promotion efforts can lose their priority. Active learning is unique in that it can simultaneously help students learn and improve their health by increasing physical activity and reducing sedentary behavior. The study goal is to understand and improve the implementation of active learning approaches, and by achieving this goal, help schools best meet the needs of their students. Developing an implementation strategy to improve the use of active learning has the potential to impact millions of children across the US and beyond.

## Data Availability

The datasets used and/or analyzed during the current study will be available from the corresponding author on reasonable request.

## Abbreviations

SCT: Social Cognitive Theory
CFIR: Consolidated Framework for Implementation Research
AIM: Acceptability of Implementation Measure
